# The Role of PDGFRA in Predicting Oncological and Immune Characteristics in Pancreatic Ductal Adenocarcinoma

**DOI:** 10.1155/2022/4148805

**Published:** 2022-03-26

**Authors:** Zijian Wu, Jin Xu, Rong Tang, Wei Wang, Bo Zhang, Xianjun Yu, Jiang Liu, Si Shi

**Affiliations:** ^1^Department of Pancreatic Surgery, Fudan University Shanghai Cancer Center, Shanghai, China; ^2^Department of Oncology, Shanghai Medical College, Fudan University, Shanghai, China; ^3^Shanghai Pancreatic Cancer Institute, Shanghai, China; ^4^Pancreatic Cancer Institute, Fudan University, Shanghai, China

## Abstract

**Objective:**

Pancreatic ductal adenocarcinoma (PDAC) is a lethal solid gastrointestinal malignancy with poor immune infiltration and a limited response to immunotherapy. The aim of our study was to explore the predictive value of platelet-derived growth factors (PDGFs) and their receptors (PDGFRs), which are widely expressed in various tumor cells.

**Methods:**

Transcriptomic data with follow-up information were obtained from the GEO, TCGA and ArrayExpress. The Kaplan–Meier (KM) method and univariate Cox (UniCox) proportional hazard regression were used to show the survival outcomes of the groups. Immune infiltration was analyzed using the online databases TISCH, TISIDB, TIMER2.0, and TIDE as well as the R packages “estimate” and “GSVA.” Mutation and functional enrichment analyses were conducted using the R packages “maftools,” “clusterProfiler,” and online repository HOME for Researchers. Finally, the results were validated in 79 samples from our cancer center.

**Results:**

Survival analysis using public databases and the FUSCC cohort indicated PDGFRA to be associated with prolonged overall survival (OS) (both *p* < 0.05). PDGFRA expression was highest in cancer-associated fibroblasts (CAFs) of PDAC, as validated in public databases and cell lines from our cancer center. The high expression of PDGFRA was associated with increased immune infiltration and potent T cell cytotoxicity in PDAC.

**Conclusion:**

In summary, high PDGFRA expression is associated with increased immune infiltration and prolonged OS. This finding might provide a new strategy for regulating immune cell infiltration in PDAC and improving the efficacy of immunotherapy.

## 1. Introduction

With a low 5-year overall survival of only 10%, pancreatic cancer, 90% of which is pancreatic ductal adenocarcinoma (PDAC), is one of the most malignant solid gastrointestinal tumors [1]. Due to the anatomical location of the cancer and the lack of early symptoms, only 10~20% of patients without distant metastasis can undergo surgical resection [2]. Despite definite progress in traditional therapies such as surgery, radiochemotherapy, and other locoregional therapies, the overall survival (OS) rate for all patients has not improved significantly. Even when a tumor is resectable, the 5-year OS rate does not exceed 20–25% [3]. Accordingly, there is an urgent need for new effective treatments for PDAC.

Compared with traditional therapies, emerging immunotherapies, including immune checkpoint inhibitor (ICI) tumor vaccines, oncolytic viruses (OVs), chimeric antigen receptor T cell (CAR-T), and other treatments targeting the tumor microenvironment (TME), have led to a therapeutic “renaissance” for several types of cancers [4, 5]. However, even groundbreaking immunotherapies have yielded limited responses in PDAC, possibly because of the unique PDAC microenvironment that contains a prominent dense, collagenous stromal compartment that forms a mechanical barrier for immune cells (CD8+ T lymphocytes, natural killer (NK) cells, and other effector cells) and inhibits their infiltration [6, 7]. Cancer-associated fibroblasts (CAFs), which are responsible for remodeling the extracellular matrix (ECM), release growth factors and other cytokines such as platelet-derived growth factor (PDGF) in an autocrine or paracrine manner to promote tumorigenesis [8]. The multiple functions of PDGF and its receptor (PDGFR), which are widely expressed in various tumor cells, have been extensively explored experimentally with regard to tumorigenesis, growth, invasiveness, and the TME [9, 10]. There are two kinds of PDGFRs, PDGFR*α* and PDGFR*β*, both of which are tyrosine kinase receptors with five extracellular immunoglobulin-like domains [11]. Antagonists targeting PDGF have been developed and applied in clinical practice over the past decades, such as the tyrosine kinase receptor inhibitors (TKIs) imatinib (used for gastrointestinal stromal tumors (GISTs)), sunitinib (used for pancreatic neuroendocrine tumors), and sorafenib (used for hepatocellular carcinoma) [12]. Overall, the relationships of PDGFR expression with the TME and tumor prognosis are worthy of investigation.

T cell infiltration is known to be associated with improved prognosis in human malignancies [13]. CD8+ T cells, also known as cytotoxic T lymphocytes (CTLs), are considered to play a prominent role in antitumor immunity once they are activated. Immune checkpoint blockade (ICB) therapies targeting programmed death 1 (PD-1) and CTL-associated antigen 4 (CTLA4) have shown promise over the past decade (13). Therefore, further prediction of anti-PD1 and anti-CTLA4 responses is a worthy pursuit. Higher CTL infiltration usually implies more antitumor immunity and therefore a better prognosis. However, immunotherapies do not always achieve such results, possibly because of mechanisms such as T cell immune evasion.

To predict the level of immune infiltration and improve the efficacy of immunotherapy for PDAC, we explored how PDGFRA expression influences the tumor immune microenvironment with data from public cancer sequencing databases and validated our findings in 79 PDAC samples from Fudan University Shanghai Cancer Center (FUSCC).

## 2. Methods

### 2.1. Patients and Datasets

Only resected clinical samples that were eventually diagnosed only as PDAC and had completed follow-up data were included. Samples from patients for whom the pathological diagnosis was neuroendocrine, cystic, mucinous, mixed, or other types of pancreatic tumors and samples from patients with insufficient clinicopathological or follow-up information were excluded. Gene expression data (E-MTAB-6134) and clinicopathological information for PDAC patients with survival outcomes were obtained from the public repository ArrayExpress (https://www.ebi.ac.uk/arrayexpress/). Transcriptomic data from The Cancer Genome Atlas (TCGA) as well as survival data for PDAC patients were obtained from the repository (https://www.cancer.gov/about-nci/organization/ccg/research/structural-genomics/tcga). We screened TCGA data with pathology information and chose data from 146 PDAC patients for further study. RNA-seq data with survival information for 125 PDAC patients from dataset GSE71729 were downloaded from the Gene Expression Omnibus (GEO) database (https://www.ncbi.nlm.nih.gov/geo/). The follow-up information for the 79 PDAC patients in the validation cohort was obtained from the Shanghai Pancreatic Cancer Institute of FUSCC. The baseline information of the two PDGFRA expression groups in these datasets was compared using SPSS, version 24.0 (SPSS, Chicago, IL, USA). Categorical variables were analyzed by Pearson's *χ*^2^ test. Continuous data are described as the means ± SDs and were analyzed by unpaired Student's *t* test.

### 2.2. Bioinformatic Algorithms

#### 2.2.1. TISCH

The Tumor Immune Single-Cell Hub (TISCH) (http://tisch.comp-genomics.org/home/) is a large-scale scRNA-seq database that enables visualization of the TME at the single-cell level across different cancer types [14]. In this study, a t-distributed stochastic neighbor embedding (t-SNE) plot of PAAD_CRA001160 and violin plots of GSE11672 and PAAD_CRA001160 were constructed in the TISCH to show PDGFRA expression in different types of immune cells in the PDAC TME.

#### 2.2.2. ESTIMATE

The tumor purity of the two groups was evaluated using the algorithm Estimation of Stromal and Immune cells in Malignant Tumors using Expression (ESTIMATE) data. This method can be used to estimate the fractions of stromal and immune cells, the nontumor cells within tumor samples, through expression levels of specific gene signatures in included samples [15]. Immune and stromal scores were calculated by using single-sample gene set enrichment analysis (ssGSEA) algorithm on the basis of unique gene signatures associated with immune cells and stromal tissues. Then, the ESTIMATE score was produced by integrating the stromal scores and the immune scores. The formula for tumor purity was listed as follows: Tumor purity = cos (0.6049872018 + 0.0001467884 × ESTIMATE score) [15].

The tumor purity, ESTIMATE, immune, and stromal scores were all calculated using the R package “estimate.”

#### 2.2.3. ssGSEA

ssGSEA was applied to analyze the immune cell infiltration in two groups with different PDGFRA levels in the PDAC expression profile from TCGA using the “GSVA” R package based on 29 immunity-associated signatures [16, 17]. Normalized ssGSEA enrichment scores were calculated for each immune category.

#### 2.2.4. TISIDB

TISIDB (http://cis.hku.hk/TISIDB/) is an integrated web repository with which users can examine interactions of certain genes with tumor immunity across different stored databases [18]. In our study, a pancancer analysis of the correlations of PDGFRA expression with CD8^+^ T cells was performed with TISIDB using multiple algorithms (TIMER, EPIC, MCPcounter, CIBERSORT, quanTIseq, and XCell). The Spearman correlations of the expression, methylation, and copy numbers of PDGFRA among 28 T lymphocyte types were also explored using TISIDB. Drugs targeting PDGFRA collected from the DrugBank database were visualized in a network diagram and in table format with TISIDB.

#### 2.2.5. TIMER2.0

TIMER2.0 (http://timer.cistrome.org/) is a web server that enables comprehensive evaluation and visualization of tumor immune infiltration with TCGA data or user-provided cancer data [19]. In this study, the correlations of PDGFRA expression with the levels of immune infiltration in diverse cancer types were visualized using TIMER2.0. The correlations of the PDGFRA expression level (log_2_ [transcripts per million, TPM]) in PDAC with tumor purity and with the level ofCD8^+^ T cell infiltration were assessed using TIMER2.0 and verified using three algorithms (TIMER, MCPcounter, and quanTIseq). The correlation between PDGFRA and CAF infiltration in the PDAC microenvironment was also analyzed with multiple algorithms (EPIC, MCPcounter, XCell, and TIDE) in TIMER2.0.

#### 2.2.6. TIDE

Tumor Immune Dysfunction and Exclusion (TIDE) (http://tide.dfci.harvard.edu) is a web platform for estimating the pretreatment robustness of particular biomarkers based on published ICB trials and nonimmunotherapy tumor profiles as well as CRISPR screens [20, 21]. The CTL infiltration level was determined according to the average expression of CTL signature genes (including CD8A, CD8B, GZMA, GZMB, and PRF1) in samples. The correlations between CTL levels and OS outcomes among different PDGFRA expression groups were calculated through the two-sided Wald test. The associations of survival outcomes with PDGFRA expression levels and potent T cell cytotoxicity in PDAC and the pancancer associations of OS with PDGFRA expression levels (displayed as Kaplan–Meier (KM) curves) were analyzed with TIDE. In the figure, the “top” and “bottom” groups refer to the groups with CTL levels above and below the average level, respectively.

### 2.3. Analysis of the Association of PDAC Mutations with PDGFRA Expression

To explore the somatic mutations in PDAC samples with different PDGFRA expression levels, somatic mutation data were obtained from the TCGA repository. The data were analyzed and visualized using the R package “maftools” (version 2.2) [22].

### 2.4. PDGFRA Functional Enrichment Analysis

Gene Ontology (GO) annotations and Kyoto Encyclopedia of Genes and Genomes (KEGG) pathway annotations were evaluated to elucidate the potential functions of PDGFRA by using the R packages “org.Hs.e.g.db” and “clusterProfiler” [23]. Three categories, the biological process (BP), cellular component (CC), and molecular function (MF) categories, were explored in the GO analysis. The results of the GO and KEGG analyses are presented in bubble charts created using the “ggplot2”package of R.

### 2.5. Analysis of the Correlations of PDGFRA with TMB and MSI

The tumor mutational burden (TMB) is defined as the total number of nonsynonymous alterations (including somatic, coding, and indel mutations and base substitutions) per megabase of genomic sequence examined using the “maftools” R package. Microsatellites (MST), also named simple sequence repeats (SSRs) or short tandem repeats (STRs), are defined as short segments (typically 2-6 base pairs) of repeating DNA uniformly distributed over the entire eukaryotic genomes. Microsatellite instability (MSI) in tumors is caused by DNA mismatch repair deficiency, a clinical marker for predicting the response to immunotherapy in different cancer types [24]. The correlations of PDGFRA expression with TMB and MSI were analyzed using the online website HOME for Researchers (https://www.home-for-researchers.com/static/index.html#/).

### 2.6. Validation of PDGFRA Expression in a Shanghai Pancreatic Cancer Institute Cohort

#### 2.6.1. Cell Culture

A normal human pancreatic duct epithelial (HPDE) cell line and seven human pancreatic cancer cell lines Panc-1, MIAPaCa-2, SW1990, CFPAC-1 AsPC-1, CAPAN-1, and BxPC-3 were acquired from the American Type Culture Collection (ATCC). PANC-1, MIAPaCa-2, and SW1990 cells were cultured in Dulbecco's modified Eagle's medium (DMEM) supplemented with 10% fetal bovine serum (FBS) and 1% antibiotics (100 U/mL penicillin, 100 *μ*g/mL streptomycin, and 0.25 *μ*g/mL amphotericin B). CFPAC-1 and CAPAN-1 cells were cultured in Iscove's modified Dulbecco's medium (IMDM) with 10% FBS and 1% antibiotics. AsPC-1 and BxPC-3 cells were cultured in Roswell Park Memorial Institute (RPMI) 1640 medium with 10% FBS and 1% antibiotics. All of the cell lines were grown in cell incubators with humidified atmospheres of 37°C and 5% CO_2_.

The CAFs in our laboratory were obtained from surgically resected human pancreatic tissues at our cancer center; the cells were purified and then immortalized [25]. First, fresh pancreatic tumor tissue was cut into 1–3 mm^3^ fragments and digested with 0.25% trypsin for 30 min at 37°C. The fragments obtained in the previous step were centrifuged at 600 × *g* for 5 min and rinsed with DMEM supplemented with 10% FBS. The tissue fragments were plated in the tissue culture dishes and allowed to adhere. The fibroblasts would then grow out of tissue fragments after they were incubated at 37°C for several days. The fibroblasts were then trypsinized and passaged for 2–3 populations until they were dissociated from the epithelial cells and then cultured in DMEM with 10% FBS and 1% antibiotics.

#### 2.6.2. RNA Isolation and Quantitative Real-Time PCR

Total RNA from tumor tissues of 79 PDAC patients who had been treated in our cancer center was extracted with TRIzol reagent (Invitrogen, Carlsbad, CA, USA). Total RNA from cells was extracted using a SteadyPure Universal RNA Extraction Kit (Accurate Biotechnology, China). cDNA was obtained by reverse transcription polymerase chain reaction (RT-PCR) using 5x Evo M-MLV Rt Master Mix (Accurate Biotechnology, China). Quantitative real-time polymerase chain reaction (qPCR) was performed using a QuantStudio 6 Flex Real-Time PCR System (Thermo Fisher Scientific, USA). The sequences of the PDGFRA primers used were as follows: human PDGFRA, 5′-TGGCAGTACCCCATGTCTGAA-3′ (forward primer) and 5′-CCAAGACCGTCACAAAAAGGC-3′ (reverse primer) and human GAPDH, 5′-GGAGCGAGATCCCTCCAAAAT-3′ (forward primer) and 5′-GGCTGTTGTCATACTTCTCATGG-3′ (reverse primer).

### 2.7. Statistical Analysis

Statistical analyses were conducted using R software version 4.0.1 and GraphPad Prism 8. The survival outcomes of E-MTAB-6134 were analyzed by univariate Cox (UniCox) proportional hazard regression. K-M curves were generated to visualize the survival outcomes of the groups, and statistical significance was compared with the log-rank test or the Gehan–Breslow–Wilcoxon test. The best cutoff points for the K-M curves were analyzed using X-tile, a bioinformatic tool for finding the best cutoff thresholds [26]. A *p* value less than 0.05 in our research was considered statistically different.

## 3. Results

### 3.1. PDGFRA Is Associated with Prolonged OS

**Figure 1:**
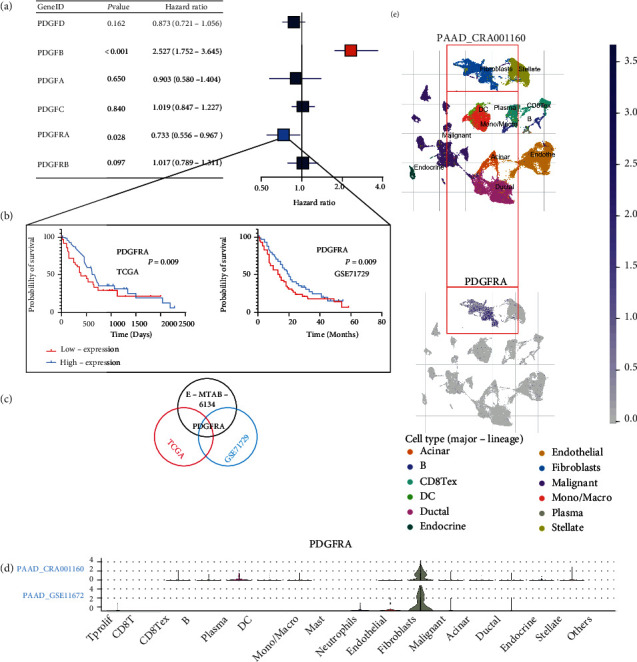
Comparison of survival outcomes and expression in different immune cell types. (a) UniCox analysis of 6 candidate genes in PDAC. (b) Prognosis illustrated by K-M curves for the PDGFRA-high and PDGFRA-low groups in both the TCGA and GSE71729 datasets. (c) Venn diagram displaying the intersection of the three datasets (E-MTAB-6134, TCGA, and GSE71729). (d) Violin plot showing the expression of PDGFRA in various immune cell types (PAAD_CRA001160 and PAAD_GSE111672). (e) t-SNE plot showing the expression of PDGFRA in different immune cells (PAAD_CRA001160).

For the selection of prognostic PDGFs or PDGFRs, 6 candidate genes, namely, PDGFA, PDGFB, PDGFC, PDGFD, PDGFRA, and PDGFRB, were included in our research. The related clinicopathological information for the E-MTAB-6134, TCGA, and GSE71729 datasets is listed in Table S1. A total of 288 adult PDAC patients from the E-MTAB-6134 dataset were included in our study. Comparison of the baseline information of the two PDGFRA expression groups showed no significant differences for any of the targets (all *p* > 0.05) (Table S2). The results of the UniCox regression test indicated that PDGFRA (*p* = 0.028) and PDGFRB (*p* < 0.001) were associated with prolonged OS in the E-MTAB-6134 cohort (Figure 1(a)). Next, comparison of the baseline information in the TCGA dataset indicated that most characteristics did not differ between the two PDGFRA groups except for the tumor location (*p* = 0.016) (Table S3). The survival outcomes of 146 PDAC patients in the TCGA dataset and 125 PDAC patients in the GSE71729 dataset were selected for analysis according to the PDGFRA expression levels. The K-M curves of the TCGA (*p* = 0.009) and GSE71729 (*p* = 0.026) data showed that high expression of PDGFRA was significantly associated with increased OS (Figure 1(b)). Therefore, we chose PDGFRA as the target gene for further analysis (Figure 1(c)). Violin and t-SNE plots revealed that PDGFRA expression was highest in CAFs in PDAC (Figure 1(d)). Overall, these results suggest that high PDGFRA expression is related to better prognosis in PDAC patients.

### 3.2. PDGFRA Is Associated with Abundant Immune Infiltration in PDAC

**Figure 2:**
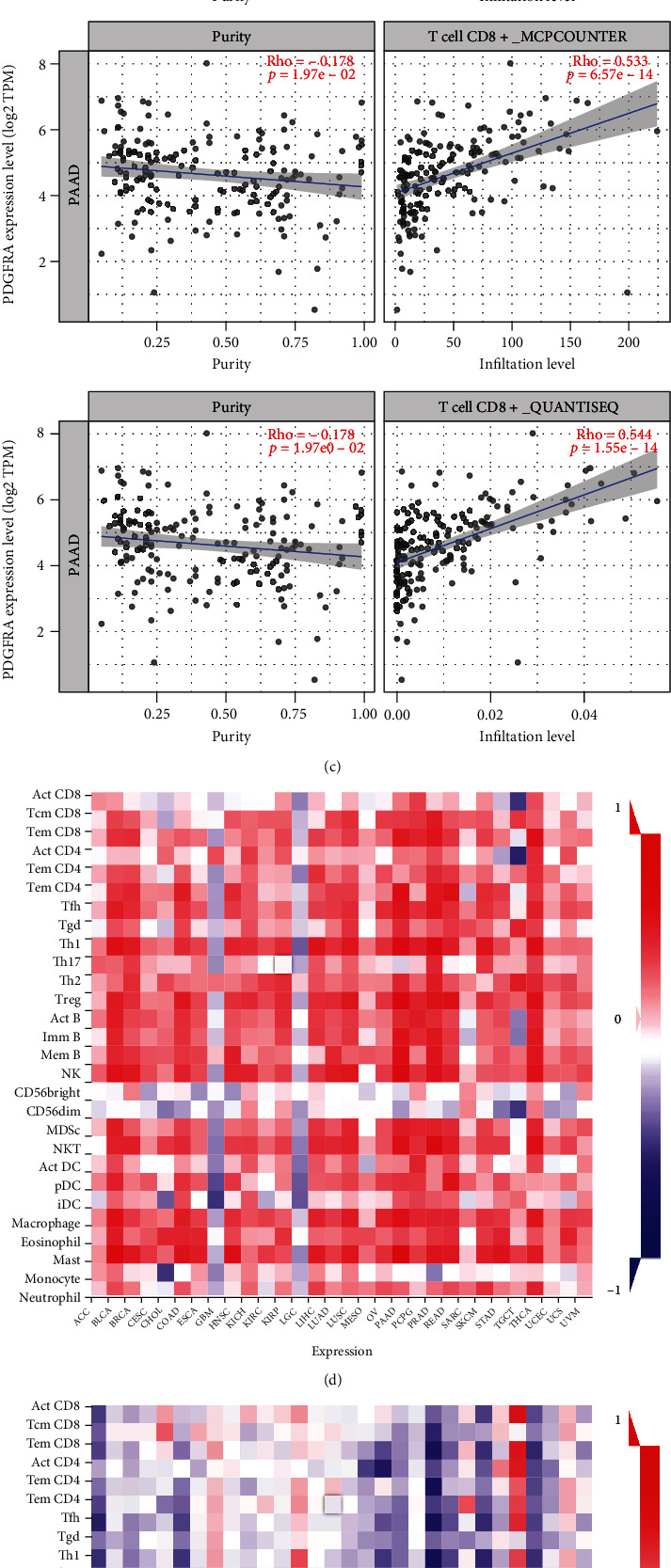
Analysis of the association of immune infiltration with PDGFRA in PDAC. (a) Heatmap showing the differences in tumor purity, ESTIMATE scores, stromal scores, and 29 immune signatures between the PDGFRA-high and PDGFRA-low groups. (b) Heatmap displaying the correlation between cancer and CD8^+^ T cells in different databases. (c) Scatterplots showing the correlations of PDGFRA expression with tumor purity and the CD8^+^ T cell infiltration level. (d–f) Heatmaps showing PDGFRA expression, methylation, and copy numbers in association with 28 T lymphocyte types.

Immune cell infiltration in TCGA PDAC samples with RNA-seq data was analyzed using the ssGSEA method. Tumor purity and ESTIMATE, immune, and stromal scores were calculated according to PDGFRA expression levels using the R package “estimate.” A heatmap showed that strong PDGFRA expression was associated with high immune cell infiltration. Similarly, annotation labels indicated that the ESTIMATE score, immune score, and stromal score of the PDGFRA-high group were greater than those of the PDGFRA-low group, though the opposite result was obtained for tumor purity (Figure 2(a)). Pancancer analysis with TIMER2.0 using 7 databases indicated that PDGFRA was significantly associated with CD8^+^ T cell infiltration in PDAC (both *p* < 0.05) (Figure 2(b)). In addition, analyses of the correlations of the PDGFRA expression level with tumor purity and CD8^+^ T cell infiltration revealed that PDGFRA expression was strongly positively associated with CD8^+^ T cell infiltration in PDAC according to TIMER (Rho = 0.771, *p* = 5.50e − 35), MCPcounter (Rho = 0.553, *p* = 6.57e − 14), and quanTIseq (Rho = 0.544, *p* = 1.55e − 14) (Figure 2(c)). The results also showed that PDGFRA expression was negatively correlated with tumor purity (Rho = −0.178, *p* = 1.97e − 02) (Figure 2(c)). Rho = 0.771 and *p* = 5.50e − 35 TISIDB was then used to explore the relationships between PDGFRA expression levels and 28 tumor immune-infiltrating cell subtypes in pancancer analysis, and the results indicated that the expression level, methylation, and copy number of PDGFRA were broadly associated with the 28 immune cell subtypes (Figures 2(d)–2(f)).

### 3.3. PDGFRA Is Associated with Potent T Cell Cytotoxicity

**Figure 3:**
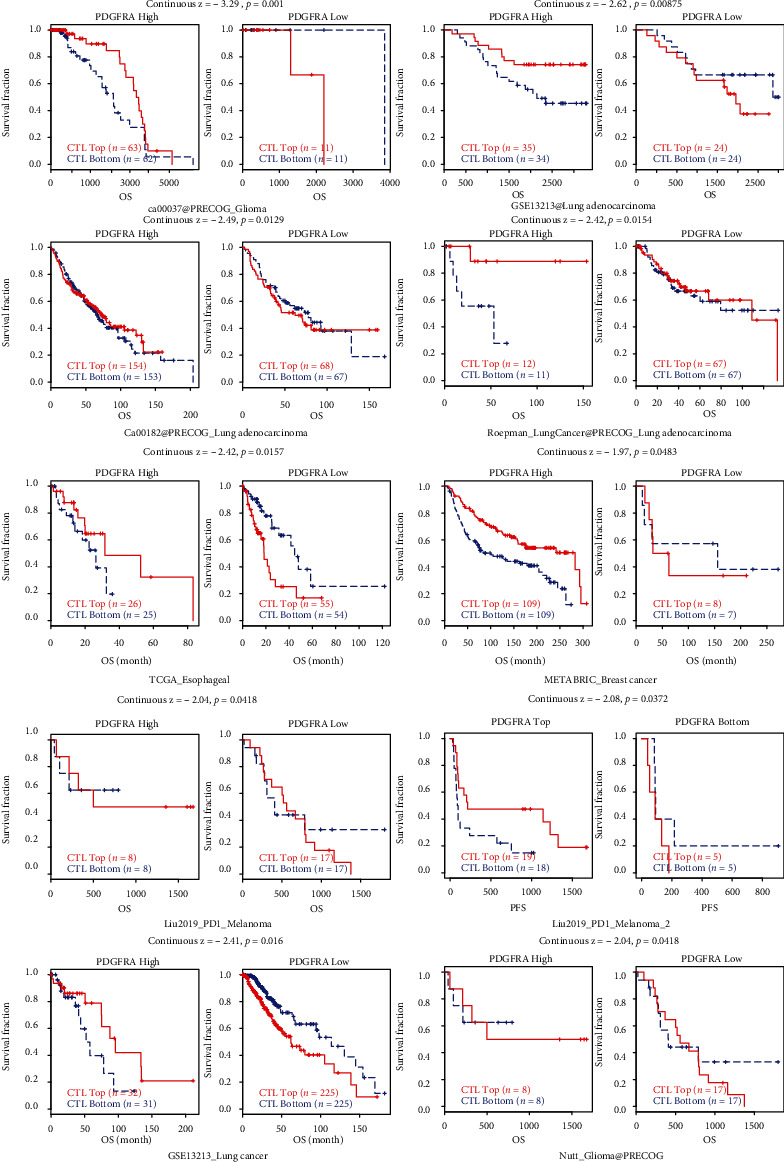
K-M curves showing the pancancer OS for both the top and bottom CTL subtypes in the PDGFRA-high and PDGFRA-low groups.

The correlation of CTLs with PDGFRA expression was analyzed using TIDE. PDGFRA was found to be significantly associated with CTLs in glioma (ca0037@PRECOG_Glioma and Nutt_Glioma@PRECOG), melanoma, lung cancer (GSE13213@Lung adenocarcinoma, ca00182@PRECOG_Lung adenocarcinoma, Roepman_LungCancer@PRECOG_Lungadenocarcinoma, and GSE13213_Lung cancer), esophageal cancer (TCGA_Esophageal), and breast cancer (METARBRIC_Breast cancer) (all *p* < 0.05) (Figure 3). However, higher CTL infiltration was associated with prolonged OS only in the PDGFRA-high group (Figure 3). Overall, high CTL infiltration resulted in better OS only in the PDGFRA-high group, which also means that anti-PD1 and anti-CTLA4 treatments should be effective in this particular group. All these results suggest that PDGFRA is a potential indicator of the immunotherapy response and that PDGFRA might regulate the cytotoxic function of T lymphocytes. However, the particular mechanisms have yet to be investigated.

### 3.4. PDAC Mutation Landscape and PDGFRA Expression

**Figure 4:**
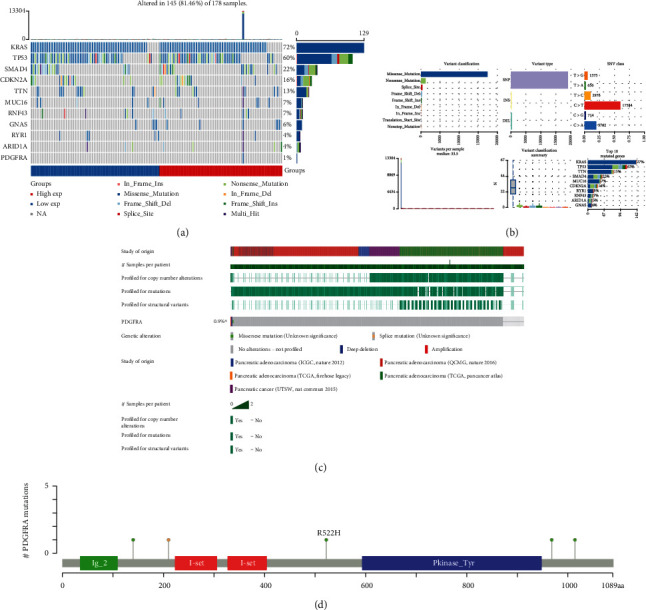
Mutation landscape of PDAC samples with high and low PDGFRA expression. (a) Oncoplots displaying the eleven most frequently mutated genes in the PDGFRA-high and PDGFRA-low groups. (b) Stacked bar charts showing the variant classifications, variant type SNV classes, variants per sample, and top mutated genes in PDAC samples. (c) Waterfall plots showing the profiles for copy number alterations, mutations, structural variants, mutation frequency, and alteration types in five datasets. (d) Lollipop plot showing the mutation distribution and protein domains of PDGFRA with hotspots labeled.

Somatic mutations in the two PDGFRA expression groups were compared using the “maftools” R package. A waterfall plot, also known as an oncoplot, indicated a similar mutation landscape for the two groups. The horizontal histogram of the oncoplot in Figure 4(a) displays the genes with the highest mutation frequencies in the two groups, including KRAS (72%), TP53 (60%), SMAD4 (22%), and CDKN2A (16%). The horizontal histogram and box diagram of variant classifications demonstrate that missense mutation was the predominant variant classification in PDAC (Figure 4(b)). The predominant variant type was single-nucleotide polymorphism (SNP), and the predominant single-nucleotide variant (SNV) class was C > T (Figure 4(b)). The numbers of variants per sample are also depicted in the histogram in Figure 4(b). Conversely, mutations in PDGFRA were scarce in PDAC; PDGFRA had a mutation rate of only 0.9% (Figure 4(c)). The mutation distribution and protein domains of PDGFRA are illustrated in a lollipop plot with labeled hotspots in Figure 4(d).

### 3.5. Enrichment Analysis of PDGFRA Expression Mainly Reveals Associations with the Extracellular Microenvironment and Immune Responses

**Figure 5:**
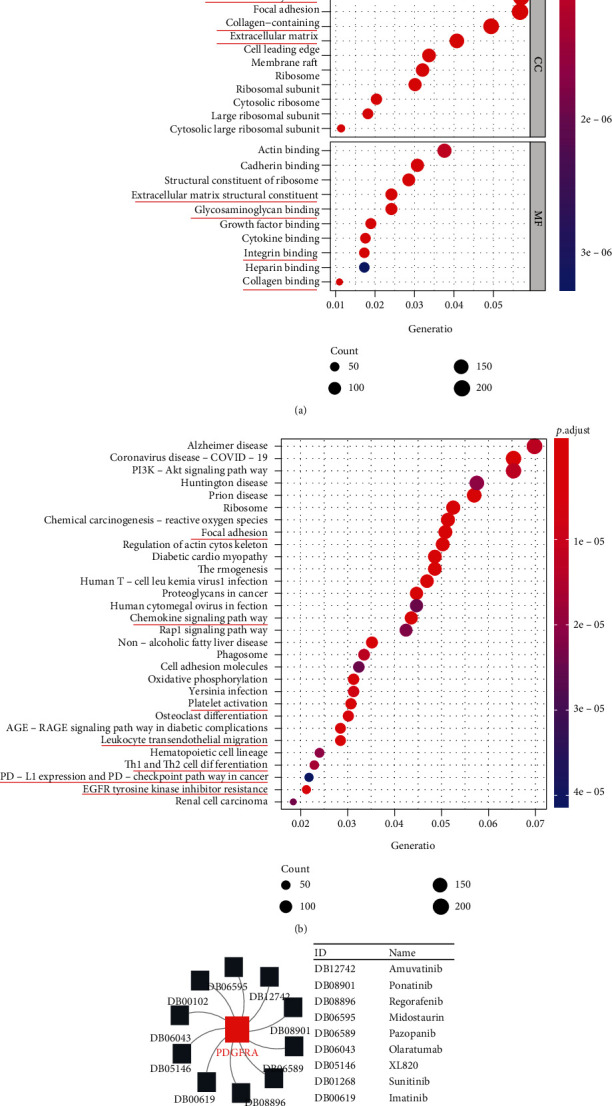
Enrichment analysis of DEGs in the PDGFRA-high and PDGFRA-low groups. (a and b) Bubble charts displaying the GO and KEGG analysis results for DEGs between the PDGFRA-high and PDGFRA-low groups. (c) Network diagram showing drugs targeting PDGFRA.

GO- and KEGG-based enrichment analysis of the differential expression genes (DEGs) in the two PDGFRA expression groups was performed. According to the bubble plots for the GO analysis, PDGFRA expression is mainly associated with ECM components such as collagen, integrin, and glycosaminoglycan and with other components in the microenvironment, with enrichment in the CC, MF, and BP categories (Figure 5(a)). The KEGG analysis bubble plots indicated that PDGFRA expression is also related to immune responses such as focal adhesion, chemokine leukocyte transendothelial migration, Th1 and Th2 cell differentiation, PDL-1 expression, PD-1 checkpoint, and EGFR TKI resistance (Figure 5(b)). Since our previous research has revealed the predictive value of PDGFRA in the immune response, investigating the drug sensitivity of PDGFRA will help elucidate the role of PDGFRA in cancer treatment. The PDGFRA-targeting drugs in the network diagram include amuvatinib (DB12742), ponatinib (DB08901), regorafenib (DB08896), midostaurin (DB06595), pazopanib (DB06589), olaratumab (DB06043), XL820 (DB05146), sunitinib (DB01268), imatinib (DB00619), and becaplermin (DB00102) (Figure 5(c)).

### 3.6. Validation of the Results in the FUSCC Cohort

To validate the results of our bioinformatic analysis, we chose eight PDAC cell lines and CAFs for PDGFRA expression level detection. Comparison of the baseline information of FUSCC cohort showed that most characteristics did not differ among the two PDGFRA groups except for that the PDGFRA low expression group is more advanced in N stage (*p* = 0.003) (Table S4). Besides, the qPCR results were consistent with the bioinformatic analysis results in that CAFs expressed a higher level of PDGFRA than other tumor cell lines (all *p* < 0.001). Regarding the prognostic value of PDGFRA, we analyzed follow-up information together with mRNA expression results for PDGFRA from our center and obtained the same result: high expression of PDGFRA was related to better survival outcomes than low expression (*p* = 0.0317).

## 4. Discussion

Pancreatic cancer is a fatal malignancy that lacks effective therapies. Neither surgery nor chemotherapy can dramatically improve the prognosis, and the disease maintains quite a low 5-year overall survival rate [2, 27–29]. Along with providing insight into the mechanisms of tumor pathogenesis, immunotherapy has achieved encouraging efficacy in multiple types of cancer, such as non-small-cell lung cancer, breast cancer, prostate cancer, and melanoma [30–33]. Compared with immunotherapy for other cancers, immunotherapy for PDAC is still in its infancy, but glimmers of promise are evident [34]. Previous studies have proposed many reasons for treatment failure, but the key contributors are the typical immunosuppressive pancreatic TME with poor immune infiltration (especially of effector T cells) and the low TMB [35, 36].

In our study, we found that patients with high PDGFRA expression had better survival outcomes than those with low PDGFRA expression, which appears to conflict with the common theme in other types of cancer in which high expression of PDGFRs is correlated with poor prognosis [37–43]. However, a study on clear-cell renal carcinoma has found that high expression of PDGFB significantly inhibits tumor growth (*p* ≤ 0.05) and decreases cancer-specific mortality (*p* ≤ 0.001) [44]. The different results might be related to pericytes, which are contractile cells of the tumor stroma that wrap around the endothelial cells of capillaries and venules throughout the body and support the formation of the blood vessels [45]. Overexpression of PDGFB promotes recruitment of pericytes, which may serve as gatekeepers against tumor invasion and metastasis [46, 47]. Furthermore, the pancancer analysis of the brain, breast, pancreatic, kidney cancer, melanoma, and lymphoma supported our conclusion (Supplementary Figure 1). These results provide value by changing the paradigm of inhibition of tumor angiogenesis in cancer treatment.

In addition to exhibiting prognostic value, our results show that PDGFRA is a qualified predictive biomarker for immune cell infiltration, especially that of effector CD8^+^ T cells, also known as CTLs. CTLs are vital executors of immune defense against intracellular pathogens, including tumor cells. After recognizing antigens and becoming activated, CD8^+^ T cells will start secreting cytokines, primarily TNF-*α* and IFN-*γ* to kill infected or malignant cells. Our pancancer analysis results indicated that increased CTL infiltration is related to prolonged OS only in samples with high expression of PDGFRA.

In our study, multiple algorithms showed that PDGFRA is highly associated with CAF infiltration in the PDAC microenvironment. However, the consensus finding is that CAFs have a predominant immunosuppressive function through IL-6, CXC motif chemokine ligand 9 (CXCL9), and TGF*β* [48]. One reason that high expression of PDGFRA with CAF infiltration leads to a better prognosis may be the heterogeneity of CAFs. Studies have reported that CAFs adjacent to tumor cells strongly express *α*-smooth muscle actin (*α*SMA); these cells are referred to as “myofibroblast CAFs (myoCAFs)”. More distal CAFs that express higher levels of IL-6 are referred to as “inflammatory CAFs (iCAFs)” [49]. The mechanisms by which these subtypes of CAFs affect tumorigenesis and the interactions between them are still under study. The outcomes of this research might alter our understanding of CAFs. A basic study focusing on stromal elements in PDAC has revealed that Hedgehog-driven stromal elements restrain tumor growth in part by antagonizing tumor angiogenesis [50]. Similarly, a clinical trial (NCT01130142) designed to deplete the tumor stroma by inhibiting Hedgehog signaling to improve immunotherapy efficacy has revealed a paradoxically worse prognosis. Research in this area is emerging, and more exploration of different CAF subtypes is needed [51].

For decades, cancers have been subtyped according to their molecular gene aberrations to potentially optimize clinical decisions. Single genetic marker for PDAC, such asCA19-9, SMAD4, and S100A2, has showed promising results for prognosis prediction in PDAC [52]. However, the single-gene research for prediction of the immune response is still insufficient, which might be the advantage of PDGFRA over multiple gene sets [53]. With regard to the potential clinical applications, PDGFRA might become a new biomarker for prediction of the immune response, especially the anti-CTLA4 response. In addition, since PDGFRA is highly expressed in CAFs and since there are already drugs targeting it, regulating the biological behavior of CAFs through PDGFRA might be a new antitumor strategy.

Some limitations of our study should be noted. First, most of the analyses in this study were conducted using public databases with bioinformatic algorithms. The mechanism by which PDGFRA regulates immune infiltration and affects prognosis is still not clear and needs to be further studied through experimental methods. Due to the limited sample size, selective bias might exist, and the results should be validated in further clinical trials.

## 5. Conclusion

In summary, the results of this research suggest that PDGFRA is able to predict immune infiltration and survival outcomes in PDAC. High PDGFRA expression is associated with increased immune infiltration and prolonged OS, which might provide a new strategy: targeting PDGFRA to regulate immune cell infiltration in PDAC.

## Data Availability

All data included in this study are available upon request by contact with the corresponding author.

## Supplementary Materials

Supplementary Materials Supplementary Figure 1: K-M curves showing the prognosis of the PDGFRA groups. High expression of PDGFRA was associated with prolonged OS in pancancer analysis with the TIDE database. Supplementary Figure 2: heatmap showing the correlation between PDGFRA expression and CAFs through multiple algorithms. The results indicate that PDGFRA is highly associated with CAF infiltration in the PDAC microenvironment. Supplementary Figure 3: scatterplots showing the relationships of PDGFRA expression with the TMB score and the MSI score in PAAD. The results indicate that PDGFRA is not related to the TMB or MSI score. Supplementary Figure 4: validation in the FUSCC cohort. (A) Histogram showing the expression of PDGFRA in various cell lines. The results indicate that PDGFRA expression is high in CAFs. (B) K-M curves showing the survival outcomes of PDGFRA groups in the FUSCC cohort. The results indicate that PDGFRA expression is related to better survival outcomes. Table S1: clinicopathological information of cohorts included. Table S2: baseline information comparison of two PDGFRA expression groups in E-MATB-6134 (*n* = 288). Table S3: baseline information comparison of two PDGFRA expression groups in TCGA (*n* = 146). Table S4: Baseline information comparison of two PDGFRA expression groups in FUSCC (*n* = 79).
