# Supplementary material for: The Role of PDGFRA in Predicting Oncological and Immune Characteristics in Pancreatic Ductal Adenocarcinoma
Source: J Oncol. 2022 Mar 26;2022:4148805. doi: 10.1155/2022/4148805 (PMC8976608; doi:10.1155/2022/4148805)
Supplement: Supplementary Materials — Supplementary Figure 1: K-M curves showing the prognosis of the PDGFRA groups. High expression of PDGFRA was associated with prolonged OS in pancancer analysis with the TIDE database. Supplementary Figure 2: heatmap showing the correlation between PDGFRA expression and CAFs through multiple algorithms. The results indicate that PDGFRA is highly associated with CAF infiltration in the PDAC microenvironment. Supplementary Figure 3: scatterplots showing the relationships of PDGFRA expression with the TMB score and the MSI score in PAAD. The results indicate that PDGFRA is not related to the TMB or MSI score. Supplementary Figure 4: validation in the FUSCC cohort. (A) Histogram showing the expression of PDGFRA in various cell lines. The results indicate that PDGFRA expression is high in CAFs. (B) K-M curves showing the survival outcomes of PDGFRA groups in the FUSCC cohort. The results indicate that PDGFRA expression is related to better survival outcomes. Table S1: clinicopathological information of cohorts included. Table S2: baseline information comparison of two PDGFRA expression groups in E-MATB-6134 (n = 288). Table S3: baseline information comparison of two PDGFRA expression groups in TCGA (n = 146). Table S4: Baseline information comparison of two PDGFRA expression groups in FUSCC (n = 79). [file 4148805.f1.zip › Supplementary Table S1-4.docx]

Table S1. Clinicopathological information of cohorts included

|  | E-MATB-6134  (n=288) | TCGA  (n=146) | GSE71729  (n=125) | FUSCC  (n=79) |
| --- | --- | --- | --- | --- |
| Sex |  |  |  |  |
| Male | 166 | 76 | 55 | 44 |
| Female | 122 | 70 | 67 | 35 |
| Age |  |  |  |  |
|  | Na | 64.88±10.95 | Na | 57.86±9.12 |
| Tumor grade |  |  |  |  |
| G1 | 110 | 19 | Na | 5 |
| G2 | 130 | 83 | Na | 46 |
| G3 | 48 | 43 | Na | 27 |
| G4 | 0 | 1 | Na | 1 |
| T stage |  |  |  |  |
| T1 | 12 | 5 | 2 | 11 |
| T2 | 47 | 15 | 20 | 50 |
| T3 | 237 | 122 | 91 | 18 |
| T4 | 0 | 3 | 1 | 0 |
| Tx | 0 | 1 | 0 | 0 |
| N stage |  |  |  |  |
| N0 | 72 | 37 | 36 | 36 |
| N1 | 216 | 108 | 80 | 43 |
| Nx | 0 | 1 |  |  |
| M |  |  |  |  |
| M0 | Na | 38 | 115 | 79 |
| M1 | Na | 107 | 2 | 0 |
| Mx | Na | 1 | Na | 0 |
| Resection margin |  |  |  |  |
| R0 | 235 | 39 | Na | 79 |
| R1 | 49 | 73 | Na | 0 |
| Rx | 4 | 43 | Na | 0 |

Table S2. **Baseline information comparison of two PDGFRA expression groups in** E-MATB-6134 (n=288).

| PDGFRA group | High (n=162) | Low (n=126) | All | *p* value |
| --- | --- | --- | --- | --- |
| Sex |  |  |  | 0.881 |
| Male | 94 (56.6) | 72 (43.4) | 166 |  |
| Female | 68 (55.7) | 54 (44.3) | 122 |  |
| Tumor grade |  |  |  | 0.105 |
| G1 | 70 (63.6) | 40 (36.4) | 110 |  |
| G2 | 65 (50) | 65 (50) | 130 |  |
| G3 | 27 (56.3) | 21 (43.7) | 48 |  |
| T stage |  |  |  | 0.405 |
| T1 | 9 (75) | 3 (25) | 12 |  |
| T2 | 22 (56.4) | 17 (43.6) | 39 |  |
| T3 | 131 (55.3) | 106 (44.7) | 237 |  |
| N stage |  |  |  | 0.681 |
| N0 | 42 (58.3) | 30 (41.7) | 72 |  |
| N1 | 120 (55.6) | 96 (44.4) | 216 |  |
| Resection margin |  |  |  | 0.396 |
| R0 | 128 (54.5) | 107 (45.5) | 235 |  |
| R1 | 31 (63.3) | 18 (36.7) | 49 |  |
| Rx | 3 (75) | 1 (25) | 4 |  |

**Notes:** By Pearson’s χ^2^ test and unpaired Student's t test; **p* values < 0.05.

Table S3. **Baseline information comparison of two PDGFRA expression groups in** TCGA (n=146).

|  | High (n=107) | Low (n=39) | All | *p* value |
| --- | --- | --- | --- | --- |
| Sex |  |  |  | 0.525 |
| Male | 54 (71.1) | 22 (28.9) | 76 |  |
| Female | 53 (75.7) | 17 (24.3) | 70 |  |
| Age | 64.26±11.12 | 66.58±10.43 |  | 0.257 |
| Tumor grade |  |  |  | 0.379 |
| G1 | 13 (68.4) | 6 (31.6) | 19 |  |
| G2 | 62 (74.7) | 21 (25.3) | 83 |  |
| G3 | 32 (74.4) | 11 (25.6) | 43 |  |
| G4 | 0 | 1 (100) | 1 |  |
| T stage |  |  |  | 0.478 |
| T1 | 3 (60) | 2 (40) | 5 |  |
| T2 | 12 (80) | 3 (20) | 15 |  |
| T3 | 90 (73.8) | 32 (26.2) | 122 |  |
| T4 | 2 (66.7) | 1 (33.3) | 3 |  |
| Tx | 0 | 1 (100.0) | 1 |  |
| N stage |  |  |  | 0.091 |
| N0 | 24 (64.9) | 13 (35.1) | 37 |  |
| N1 | 83 (76.9) | 25 (23.1) | 108 |  |
| Nx | 0 | 1 (100.0) | 1 |  |
| M stage |  |  |  | 0.098 |
| M0 | 54 (81.8) | 12 (18.2) | 66 |  |
| M1 | 3 (75) | 1 (25) | 4 |  |
| Mx | 50 (65.8) | 26 (34.2) | 76 |  |
| Tumor location |  |  |  | 0.016* |
| Head | 5 (45.5) | 6 (54.5) | 11 |  |
| Body | 80 (74.1) | 28 (25.9) | 108 |  |
| Tail | 6 (54.5) | 5 (45.5) | 11 |  |
| Overlapping | 2 (100) | 0 | 2 |  |
| Pancreas | 14 (100) | 0 | 14 |  |
| Resection margin |  |  |  | 0.835 |
| R0 | 23 (76.7) | 7 (23.3) | 30 |  |
| R1 | 52 (71.2) | 21 (28.8) | 73 |  |
| Rx | 32 (74.4) | 11 (25.6) | 43 |  |

**Notes:** By Pearson’s χ^2^ test and unpaired Student's t test; **p* values < 0.05.

Table S4. **Baseline information comparison of two PDGFRA expression groups in** FUSCC (n=79).

|  | High (n=42) | Low (n=37) | All | *p* value |
| --- | --- | --- | --- | --- |
| Sex |  |  |  | 0.783 |
| Male | 24 (54.5) | 20 (45.5) | 44 |  |
| Female | 18 (51.4) | 17 (48.6) | 35 |  |
| Age | 58.17±8.95 | 57.5±9.42 |  | 0.753 |
| Tumor grade |  |  |  | 0.411 |
| G1 | 4 (80) | 1 (20) | 5 |  |
| G2 | 25 (54.3) | 21 (45.7) | 46 |  |
| G3 | 13 (48.1) | 14 (51.9) | 27 |  |
| G4 | 0 | 1 (100) | 1 |  |
| T stage |  |  |  | 0.150 |
| T1 | 6 (54.5) | 5 (45.5) | 11 |  |
| T2 | 30 (60) | 20 (40) | 50 |  |
| T3 | 6 (33.3) | 12 (66.7) | 18 |  |
| N stage |  |  |  | 0.003* |
| N0 | 24 (66.7) | 12 (33.3) | 36 |  |
| N1 | 18 (51.4) | 17 (48.6) | 35 |  |
| N2 | 0 | 8 (100) | 8 |  |
| M stage |  |  |  |  |
| M0 | 42 (50) | 37 (50) | 79 |  |
| M1 | 0 | 0 | 0 |  |
| Mx | 0 | 0 | 0 |  |
| Tumor location |  |  |  | 0.087 |
| Head | 18 (43.9) | 23 (56.1) | 41 |  |
| Body-tail | 24 (63.2) | 14 (36.8) | 38 |  |

**Notes:** By Pearson’s χ^2^ test and unpaired Student's t test; **p* values < 0.05.
